# Transcriptomic Context of *RUNX3* Expression in Monocytes: A Cross-Sectional Analysis

**DOI:** 10.3390/biomedicines11061698

**Published:** 2023-06-13

**Authors:** Emilia Dybska, Jan Krzysztof Nowak, Jarosław Walkowiak

**Affiliations:** Department of Pediatric Gastroenterology and Metabolic Diseases, Poznan University of Medical Sciences, 60-572 Poznan, Poland; emilia.dybska@student.ump.edu.pl (E.D.); jan.nowak@ump.edu.pl (J.K.N.)

**Keywords:** monocyte, *RUNX3* expression, transcriptome, immunity, inflammation

## Abstract

The runt-related transcription factor 3 (RUNX3) regulates the differentiation of monocytes and their response to inflammation. However, the transcriptomic context of *RUNX3* expression in blood monocytes remains poorly understood. We aim to learn about *RUNX3* from its relationships within transcriptomes of bulk CD14+ cells in adults. This study used immunomagnetically sorted CD14+ cell gene expression microarray data from the Multi-Ethnic Study of Atherosclerosis (MESA, n = 1202, GSE56047) and the Correlated Expression and Disease Association Research (CEDAR, n = 281, E-MTAB-6667) cohorts. The data were preprocessed, subjected to *RUNX3*-focused correlation analyses and random forest modeling, followed by the gene ontology analysis. Immunity-focused differential ratio analysis with intermediary inference (DRAIMI) was used to integrate the data with protein–protein interaction network. Correlation analysis of *RUNX3* expression revealed the strongest positive association for *EVL* (r_mean_ = 0.75, p_FDR-MESA_ = 5.37 × 10^−140^, p_FDR-CEDAR_ = 5.52 × 10^−80^), *ARHGAP17* (r_mean_ = 0.74, p_FDR-MESA_ = 1.13 × 10^−169^, p_FDR-CEDAR_ = 9.20 × 10^−59^), *DNMT1* (r_mean_ = 0.74, p_FDR-MESA_ = 1.10 × 10^−169^, p_FDR-CEDAR_ = 1.67 × 10^−58^), and *CLEC16A* (r_mean_ = 0.72, p_FDR-MESA_ = 3.51 × 10^−154^, p_FDR-CEDAR_ = 2.27 × 10^−55^), while the top negative correlates were *C2ORF76* (r_mean_ = −0.57, p_FDR-MESA_ = 8.70 × 10^−94^, p_FDR-CEDAR_ = 1.31 × 10^−25^) and *TBC1D7* (r_mean_ = −0.55, p_FDR-MESA_ = 1.36 × 10^−69^, p_FDR-CEDAR_ = 7.81 × 10^−30^). The *RUNX3*-associated transcriptome signature was involved in mRNA metabolism, signal transduction, and the organization of cytoskeleton, chromosomes, and chromatin, which may all accompany mitosis. Transcriptomic context of *RUNX3* expression in monocytes hints at its relationship with cell growth, shape maintenance, and aspects of the immune response, including tyrosine kinases.

## 1. Introduction

Circulating monocytes belong to a heterogeneous population of myeloid cells, which originate in the bone marrow and share a common progenitor with neutrophils. Major subsets include classical CD14+CD16- and the proinflammatory CD14+CD16+ monocytes. Both types express a C-C motif chemokine receptor 2 (CCR2), which determines the egress from the bone marrow [1]. Circulating growth factors, proinflammatory cytokines, and microbial products direct further monocyte trafficking via the bloodstream to peripheral tissues. Monocyte recruitment guided by chemokines takes place during both homeostasis and inflammation and involves the activity of runt-related transcription factor 3 (*RUNX3*), which is the focus of this work.

Macrophage (M) differentiation from monocytes strengthens innate immunity within tissues [2]. Specifically, M2 macrophages support wound healing through the synthesis of interleukin-10 (IL-10). IL-10 limits the host’s immune response to pathogens and contributes to tissue repair. In contrast, M1 macrophages’ activity is proinflammatory [1] and may fuel autoimmune disease by dysregulating adaptive immunity through sustained inflammation. A deficiency of pro-resolving monocyte/macrophage functions may contribute to pathology such as inflammatory bowel disease (IBD) [3]. IBD refers to chronic inflammation of the digestive tract that manifests most commonly as Crohn’s disease (CD) and ulcerative colitis (UC), which in highly developed countries affect 0.3–1% of population. IBD results from abnormal immune responses that develop in genetically susceptible individuals when exposed to environmental risk factors [1,2], and RUNX3 deficiency has been linked to IBD development [4,5]. Moreover, a new transcriptomic prognostication marker in IBD contains genes relevant to monocyte/macrophage polarization [6]. The classical distinction between M1 and M2 macrophages does not reflect the full diversity of macrophage specialization but underscores an important inflammation-related dichotomy. Characteristics of M1 and M2 polarization can be found in circulating CD14+ cells, which constitute a mixture of various states. This study regards an analysis of such heterogenous, bulk CD14+ cell sets, to better understand *RUNX3*.

Circulating monocytes constantly replenish and maintain the macrophage population in the intestine. CCR2 and β7-integrin signaling provide balanced monocyte homing in gut homeostasis. However, protection against invading bacteria or viruses requires an increase in the frequency of inflammatory monocyte migration, which occurs only in a CCR2-dependent manner. Enhanced CCR2-mediated recruitment may be detrimental and cause immunopathology due to phenotypic and functional changes in myeloid cells [7]. Migration of nonclassical monocytes via α4β7 integrin may, in turn, support tissue healing but is unintendedly reduced by the use of the anti-α4β7 monoclonal antibody, the IBD medication vedolizumab [8]. The net result of vedolizumab in IBD is beneficial, but this limitation highlights the practical value of understanding monocyte biology for IBD management.

One of the IBD suppressibility loci, associated with myeloid cell differentiation and migratory traits via changes in chromatin structure, is mapped within *RUNX3*. RUNX3 contains an evolutionarily conserved Runt domain (which binds DNA and proteins) and belongs to key regulators of hematopoiesis and specific immune-cell lineage commitment [9,10]. Its autonomous function orchestrates monocyte extravasation [11] and maturation of colonic anti-inflammatory mononuclear phagocytes [12]. Macrophages embedded in intestinal tissue show high expression of *Runx3* [13], which may be related to their antigen-presenting capacity [2]. The loss of *Runx3* may contribute to leukocytic infiltration and the spontaneous development of colitis at an early age [10], but it is unclear whether this results from the loss of *Runx3* in monocytes, T cells, dendritic cells, or NK cells, of which especially the latter are known to strongly express *Runx3*. Overall, *RUNX3* is a gene clearly playing a role in immunity that warrants a fundamental study in the context of autoimmunity, going beyond the traditionally explored context of *RUNX3* and gastrointestinal oncogenesis.

Gene expression profiling has helped to understand gene regulation and interrelationships and to delineate immune cell subpopulations and trace their development. However, *RUNX3* has not been subject to a focused transcriptomic overview in monocytes. This may be because a typical transcriptomic pipeline involves a comparison of two groups as determined by the experimenter. Moreover, *RUNX3* is more often studied in oncology than immunity, and the evidence of *RUNX3* roles is rare. Publicly available rich datasets enable us to change this by specifically learning about *RUNX3*. Here, we aim to determine the correlates of *RUNX3* to discover new roles of the *RUNX3* gene, with a focus on immunity, using transcriptomic profiles of CD14+ cells from the blood.

## 2. Materials and Methods

**Figure 1:**
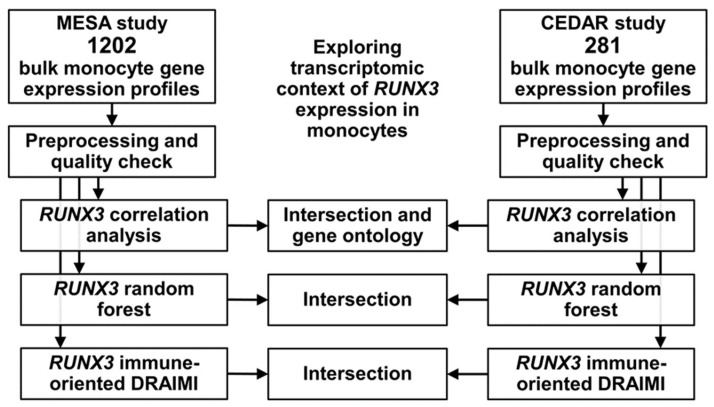
Scheme of this study.

This study was based on data made publicly available by Liu et al. [14], Bild et al. [15], and Momozawa et al. [16] as a result of two large projects. The Multi-Ethnic Study of Atherosclerosis (MESA) was a population-based study of subclinical cardiovascular disease in 6814 asymptomatic Americans aged 45–84 years. It integrated epigenomic and transcriptomic data from immunomagnetically separated human monocytes (Gene Expression Omnibus accession GSE56047). In MESA, venous blood from 1264 subjects (randomly selected) was sampled to heparin tubes, and peripheral blood mononuclear cells (PBMCs) were isolated, from which CD14+ cells were obtained with the positive immunomagnetic method (Miltenyi Biotec, Bergisch Gladbach, Germany). Median RNA integrity number (RIN) was high (9.9), and only samples with high RIN of >9.0 were subject to expression profiling with microarray in MESA. The Correlated Expression and Disease Association Research (CEDAR) was conducted in over 323 Europeans, of whom over 85% were healthy, and only subjects labeled as such were included in this analysis. Participants aged 19–86 years were included in CEDAR to study gene expression profiles in immunomagnetically separated leukocytes and mucosal biopsies from the colon and ileum. CD14+ peripheral blood monocytes were also obtained through PBMC isolation in a density gradient and positive immunomagnetic separation (BioStudies accession E-MTAB-6667). Gene expression profiling in MESA and CEDAR was performed using the HumanHT 12 v4.0 Gene Expression BeadChip (Illumina, San Diego, CA, USA). Data from MESA and CEDAR were read from public repositories, and after a quality check, normalization and log-transformation, downstream analyses were performed. Of note, MESA included adults from the general adult population, excluding participants with serious medical conditions. Therefore, data obtained in healthy adults from CEDAR and general adult population from MESA were subjected to *RUNX3*-focused analyses following the study design summarized in Figure 1.

Pearson correlation coefficients for associations between *RUNX3* and all the other transcripts, along with *p*-values, were calculated using *cor.test*. Random forests to predict *RUNX3* expression were performed within *caret* by employing *ranger*. Five repeats of 10-fold cross-validation were run. The mtry values were between 5 and 50 in the increments of 5, the splitrule was extratrees, and minimum node size was set to 2 or 3, to prevent overfitting. The number of trees was 101. Variable importance was calculated from impurity.

Immunity-focused Differential Ratio Analysis with InterMediary Inference (DRAIMI) was performed using in-house scripts as described in our previous work [6]. In brief, DRAIMI identifies top transcript ratios most consistently differing between two groups via a bootstrap procedure and identifies potential pivot genes on the grounds that they exhibit direct protein–protein interaction (STRING-DB) with both proteins encoded by transcripts involved in a ratio. Because of computational limitations, the analysis is limited to a set of pathways of interest. In this study, almost the entire Reactome immunity gene set (2112 genes, of which 1802 were present in MESA, 85.3%, and 1715 in CEDAR, 81.2%) was taken as a starting point. This was demonstrated to provide mechanistically plausible targets that are not found with differential expression analysis. DRAIMI was used to compare samples from the upper and bottom decile of *RUNX3* expression. Of note, this study does not include the differential expression analysis of the upper vs. bottom decile because it would yield results redundant with the correlation analysis.

Gene ontology was investigated with biological processes from Gene Set Enrichment Analysis (MSigDB at the Broad Institute, https://www.gsea-msigdb.org/gsea/msigdb (accessed on 15 February 2023)) and PANTHER pathways (http://pantherdb.org (accessed on 15 February 2023)) A protein–protein interaction network was built and clustered using STRING web interface.

This study did not require a bioethical approval.

## 3. Results

The mean age of MESA participants, representative of general adult population without serious illness, was 60.2 years (±9.4 y, IQR 52.0–68.0 y, 44–83 y). In MESA, 1202 bulk monocyte gene expression profiles were generated. The number of genes with median expression greater than zero across the dataset was 14,801.

We also included data from 281 healthy CEDAR participants who had a mean age of 54.7 y (±13.2 y, IQR 48–64 y, 17–82 y), and of whom 159 were female and 122 male; 218 were non-smoking and 63 smoking. In CEDAR, we used a median expression threshold of 1, which was reached by 15,136 probes.

After adding offset to both MESA and CEDAR transcriptomes, the data were log-transformed. MESA an CEDAR data were preprocessed and analyzed separately.

### 3.1. Correlation Analysis and Gene Ontology

Genes most strongly correlated with *RUNX3* in monocytes from both MESA and CEDAR studies included *EVL*, *ARHGAP17*, and *DNMT1* (Table 1). Entities top negatively correlated with *RUNX3* included two transcripts of unknown significance and *TBC1D7*. There was a considerable overlap between top results across MESA and CEDAR cohorts (Supplementary Table S1).

Some of the most known immunity genes among positive corelates in MESA, replicated in CEDAR (all mean p_adj_ < 10^−17^), included *TYK2* (r_mean_ = 0.60), *JAK1* (r_mean_ = 0.59), *PLCG2* (r_mean_ = 0.59), *IL18BP* (r_mean_ = 0.55), *IKBKB* (r_mean_ = 0.55) and *IKBKG* (r_mean_ = 0.50), *IL4R* (r_mean_ = 0.55), *SMAD3* (r_mean_ = 0.54), *LILRB1* (r_mean_ = 0.52), *ITGAL* (r_mean_ = 0.51), *IRAK1* (r_mean_ = 0.52), *OAS2* (r_mean_ = 0.49), *IRF7* (r_mean_ = 0.49), *INFAR1* (r_mean_ = 0.48), *IL16* (r_mean_ = 0.46), *IRF1* (r_mean_ = 0.47), *IL10RA* (r_mean_ = 0.42), and *ITGA4* (r_mean_ = 0.41).

The gene ontology analysis of the main transcripts positively associated with *RUNX3* revealed their association with mRNA metabolism, signal transduction, and the organization of cytoskeleton, chromosomes, and chromatin, which may all accompany mitosis. Negative correlates of *RUNX3* in monocytes enriched only a few terms, which centered on mitochondria and protein transport (Table 2).

### 3.2. Random Forest Feature Selection

Random forest models built to predict *RUNX3* expression using other transcripts achieved good performance. MESA random forest model employed mtry value of 45 and minimum node size of 2 with the extratrees splitrule, providing R^2^ = 0.562 (mean average error 0.249). CEDAR model used an mtry of 50 and a minimum node size of 3, yielding R^2^ = 0.719 and a mean average error of 0.316. Genes with the greatest predictive power considerably differed between MESA and CEDAR, as could be expected of this feature selection method (Table 3). Few genes with known immune functions were included (*TNFRSF1B, NLRP1, CEBPB, STAT4*), of which some were suggestive of SMAD activity (*MAPK7, FURIN, CCNK, CCNC*) or the inflammasome (*NLRP3, CASP1, TXN*; Supplementary Table S2).

Only *ARHGEF18* (rho/rac guanine nucleotide exchange factor 18) was selected by both random forest models with importance > 70. Further intersection of genes with importance > 40 yielded *ARHGAP17*, along with *SLC9A1* (solute carrier family 9 member A1) and *TACC1* (transforming acidic coiled-coil-containing protein 1, Table 4), providing links to cytoskeleton dynamics and signal transduction. All genes included in the models, together with impurity-derived importance, are presented in Supplementary Table S2.

### 3.3. Immune-Oriented DRAIMI

Comparison of samples from upper and lower *RUNX3* expression deciles using DRAIMI yielded results consistent across MESA and CEDAR cohorts (Table 5). The first gene on the list, *NGF* (nerve growth factor), is related to cytoskeleton dynamics, but is also known to be associated with allergic rhinitis. Other key results included well-known immune factors, protooncogenes, and some thought-inspiring findings, such as *VLDLR* (very low-density lipoprotein receptor). A bias of results towards genes with immune functions was expected because of the focus of analysis. Complete DRAIMI results are presented in Supplementary Table S3.

DRAIMI is a protein–protein interaction network-based script, and therefore the top results (n = 100) were overlaid back onto a protein–protein interaction network. The resulting graph (Supplementary Figure S1) enabled the identification of three main clusters: mitotic, immune/cell-cycle-related, and associated with antigen presentation. RUNX3 was a member of the second of these clusters, in a location suggesting functions related to both the cell cycle and immunity. The gene ontology analysis of the 20 most strongly intermediating genes indicated the following processes: transmembrane receptor protein tyrosine kinase signaling pathway (overrepresentation 24.5×, p_FDR_ = 3.37 × 10^−8^), cell surface receptor signaling pathway (6.4×, p_FDR_ = 1.87 × 10^−5^), positive regulation of intracellular signal transduction in the MAPK pathway (10.24×, p_FDR_ = 2.46 × 10^−5^), and in the ERK1/2 cascade (6.56×, p_FDR_ = 7.33 × 10^−5^), negative regulation of synaptic vesicle exocytosis (>100×, p_FDR_ = 6.00 × 10^−5^), T-cell co-stimulation (>100×, p_FDR_ = 7.01 × 10^−5^), neurotrophin signaling (>100×, p_FDR_ = 6.96 × 10^−4^), and small GTPase-mediated signal transduction (15.90×, p_FDR_ = 0.01).

## 4. Discussion

Monocytes are white blood cells derived from bone marrow hematopoietic progenitors. After entering the bloodstream, circulating monocytes become key players that recognize danger molecules via pattern recognition receptors. Moreover, cell CCR2-dependant transition to morphologically and functionally heterogeneous effector begins within the vasculature. Marginating pools have up to three days to acquire traits of immature macrophages. Gene expression changes during the monocyte-to-macrophage transition and microbial infections have been previously analyzed in distinct populations of monocytes [17,18]. However, the global gene expression profile in circulating monocytes, especially in *RUNX3*-related immune regulation, is not well characterized. To better understand the biology of monocytes, we applied three analytical approaches to *RUNX3*-focused transcriptome profiling in healthy controls from the CEDAR and the general population from MESA (Figure 1). Our results provide a list of genes likely relevant for *RUNX3*-associated myeloid pool maintenance. Representatives coupled with *RUNX3* were predominantly involved in transcription regulation (*DNMT1, TACC1*), cytoskeleton dynamics (*EVL*, *ARHGEF18*, *NGF),* and signal transduction (*ARHGAP17*, *SLC9A1*, *TBC1D7*). Thought-stimulating correlations referred to mitochondria (*CHCHD1*) and lipid accumulation (*VLDLR*).

### 4.1. Transcriptional Control of Monocyte Development

Our results confirmed that among analyzed genes from MESA and CEDAR cohorts, more than 7000 correlates moved in the same direction with *RUNX3* in monocytes. One of the strongest positive correlations belonged to DNA methyltransferase 1 (*DNMT1*). The RUNX3 is a transcription factor that modulates the effector program in the myeloid lineage [19]. Methyl groups frequently target the *RUNX3* site, which may decrease gene expression in the mammalian genome. Maintaining DNA methylation depends on copying the preexisting methylation hallmarks onto a newly replicated DNA strand. The site-specific process undergoes Dnmt1 control [20]. Previous studies, gave prominence to DNMT1-mediated methylation in inflammation and carcinogenesis. Methyltransferase controlled the methylation status of peroxisome proliferator-activated receptor gamma (PPAR-γ). The DNMT1 reduced PPAR-γ, a key suppressor of NF-κB-directed proinflammatory responses. It intensified the synthesis of proinflammatory cytokines and elevated the abundance of CD14+CD16+ monocytes in the vascular system [21]. This provides a putative link between the RUNX3/NF-κB axes. On the other hand, a report on methylated brain expressed X-linked 1 (*BEX1*) brought our attention to the Wnt/β-catenin pathway. Wang et al. showed that *DNMT1*-mediated reduction in BEX1 expression released RUNX3 to downregulate β-catenin transcription, which led to the inhibition of Wnt/β-catenin signaling in non-cancerous tissues [22].

To better understand the transcriptome landscape, we extracted genes with the highest importance (above 40 in the random forest variable importance analysis) for predicting *RUNX3* expression. Among four genes that overlapped between the two datasets, transforming acidic coiled-coil containing protein 1 (*TACC1*) contributed to transcription regulation in monocytes. Except for cytokinesis-related functions, TACC proteins interact with CBP/p300 and provide a scaffold for transcriptional complexes around nuclear receptors for retinoids. Thus, TACC1 acts as an essential coactivator of retinoic acid receptor alpha (RARα) [23]. These observations suggest a putative link with *RUNX3* that nuclear localization and transcriptional activity depend on interactions with CBP/p300 [24]. Moreover, Runx proteins act downstream of RA and TGF-β1 [25]. Interestingly, *TACC1* association with the *RUNX* family occurred in cancer of the myeloid line. TACC domain induced the *RUNX1-TACC1* fusion that resulted in myeloid leukemogenesis [26]. Knowing that RUNX3 shows similar DNA binding activity to RUNX1, it leaves the field to further investigation.

### 4.2. Cytoskeletal Structure for Monocyte Survival

The cytoskeletal remodeling is essential to support myeloid cell functions, shape, and internal organization. During hematopoiesis, expression of *RUNX3* mRNA decreases with granulopoiesis but remains stable in monocytic differentiation [9]. Thus, we explored the importance of *RUNX3* partners involved in cytoskeletal network dynamics and made a ranked list of co-expression in monocytes. We observed the strongest positive correlation for the enah/vasp-like gene (*EVL*), coding actin-associated proteins. The *EVL* has been previously described as a host of microRNA-342, having its expressions coordinated [27]. The antagonistic interaction between gene and miRNA determined specific hematopoietic lineage commitment. Overexpression of Evl-elicited lymphopoiesis, whereas *miR-342* induced myelopoiesis in vitro and in vivo [28]. Especially high expression of *miR-342* characterized proinflammatory CD16+ monocytes [29]. Interestingly, genes targeted by *miR-342* were also determined as individual sponges, suppressing the *miR-342* function during myeloid colony formation [28]. Similarly, overexpression of RUNX3 caused transcriptional repression of myeloid genes, limiting human myelopoiesis [9]. Alterations in *RUNX3-EVL*/*miR-342* axis were commonly showed in human pathologies. Methylation of CpG islands was one of the mechanisms that enabled the silencing of *RUNX3* and *EVL/hsa-miR-342* loci, characterized as an early event in colorectal carcinogenesis [27,30]. Contrarily, sustained *EVL* and *RUNX3* overexpression was associated with lymphoid [28] and myeloid leukemia occurrence [9], respectively.

We supplemented the correlation analysis with *RUNX3* expression-predicting random forest to identify genes with the highest importance in MESA and CEDAR intersection. Top place was taken by rho/rac guanine nucleotide exchange factor 18 (*ARHGEF18*). The protein encoded by this gene directly controls rho GTPases activation and acts as inductor of actin stress fibers formation [31]. Actin polymerization is regulated by the switch between the GTP to the GDP. This process, mediated by small GTPases of the Rho family, stabilizes leukocyte adhesion and improves cell resistance to deformation. Several studies have established the role of actin cytoskeleton genes for cell dynamic response in mitogen-activated protein kinase (MAPK) signaling cascade with its downstream nuclear factor kappa B (NFκB) [32,33,34]. Because MAP3K7 is a central kinase in the pathway, the knockout of *MAP3K7* and the interactor *ARHGEF18* partially reduces NFκB activity in monocytes [33]. The NF-κB pathway promotes the expression of pro-inflammatory genes and to some extent induces expression of Runx3. Considering possible protein–protein link in a cell functional downstream phenotype, activation of NF-κB pathway and inflammatory cytokine production might also be reversed by upregulation of Runx3 [35,36].

Remodeling of the actin cytoskeleton appears to activate the MAPK pathway and the pro-inflammatory characteristics of adherent myeloid cells [32]. Here, we incorporated large-scale datasets to find other mediators within a protein–protein interaction network. Of these involved in intracellular organization, nerve growth factor (*NGF*) was strongly associated with *RUNX3*. The differentiated cellular state and functional activity condition the baseline NGF requirement of the human monocyte. NGF can exhibit either pro-inflammatory or anti-inflammatory effects [37]. As a nervous-immune system cross-talk, the immature NGF precursor (proNGF) drives rearrangement in the actin structures, activating neuronal apoptosis. Mature *NGF* provides survival phenotype through TrkA regulation [38]. Consistent with the expression of NGF, circulating monocytes display the expression of neurotrophins and their specific tyrosine kinases receptors (high-affinity TrkA-C) and tumor necrosis factor receptor (low-affinity p75) [38,39,40]. NGF specifically interacts with TrkA triggering signals to activate survival AKT or differentiation MAPK downstream cascades. In turn, Runx transcription factors regulate the expression of neurotrophin receptors. Although Runx3 mainly promotes a TrkC, murine models provided explanation of diminished TrkA in animals lacking Runx3 [41]. These suggested positive stimulation of Runx3 on both TrkA and TrkC expression, giving empirical evidence to support our findings.

### 4.3. Signal Transduction in Monocytes

Comparison of genes over- and underexpressed in a given cluster with their enrichment in gene ontology terms allowed us to identify an association between *RUNX3* and the genes involved in GTPase activation. The purposed interaction network included stimuli from rho GTPase activating protein 17 (*ARHGAP17*) involved actin filament reorganization, TBC1 domain family member 7 (*TBC1D7*) controlling mitochondrial oxidative stress, and solute carrier family 9 member A1 (*SLC9A1*) managing pH regulation. Changes in *ARHGAP17* expression positively correlated with *RUNX3* in mononuclear phagocytes. Both in vitro and in vivo experiments confirmed the protective role of *ARHGAP17* for gut permeability. Arhgap17-deficient mice are known for enhanced permeability and aberrant tight junction in the gut without colitis [42]. Contrary, Runx3 KO animals spontaneously develop IBD with early onset and leukocyte infiltration [10,12]. Mechanically, the Wnt signaling pathway contributed to the functions of ARHGAP17 in colon disorders. As an expression of β-catenin inversely correlated with *ARHGAP17*, inhibition of the Wnt pathway in *ARHGAP17* knockdown cells attenuated the tumors’ promotion [43]. In this regard, *RUNX3-ARHGAP17* genes and the Wnt pathway appear to work in feedback loops. 

On the other hand, we found that the expression of *TBC1D7* significantly increased as a signal from *RUNX3* decreased, and vice versa. This was noted at the top of our negative correlations list in circulating monocytes. The protein encoded by the *TBC1D7*, together with TSC1 and TSC2, creates a complex orchestrating negative regulation of the mammalian target of the rapamycin complex 1 (mTORC1) signaling cascade. Mechanistically, the proper TSC–TBC complex plays a GTPase-activating protein for Ras homolog (RHEB), a key activator in the pathway. Changes in TBC1D7 expression may disrupt the formation of the TSC complex, leading to the intensification of mTORC1 signaling. This affects protein translation, especially by specific oxidation-reduction potential or aberrant sensing of growth factors [44]. The cumulation of reactive oxygen species may also trigger Notch1. Despite the Notch1 role in ROS level reduction [45], its signaling also regulates immunity through monocyte to M1 macrophage differentiation and RUNX3 induction [46]. Knowing that *RUNX3* represses *TBC1D7,* our observation appears to pinpoint *RUNX3* to reciprocal control of the Wnt/β-catenin and PI3K/AKT/mTORC1 pathways, inter alia involved in the failure of colorectal cancer treatment [47].

Additionally, the “wisdom of the crowd” approach with an actual random forest model allowed us to place *SLC9A1* in *RUNX3*-dependent cellular metabolism reprogramming in monocytes. Solute carrier family 9 member A1 (*SLC9A1*), also known as *NHE1*, codes a transmembrane ion transporter that exchanges intracellular H+ for extracellular Na+. Gene plays a housekeeper of cell volume, level of ROS and pH derived from redox reactions, and mitochondrial pathway of apoptosis in the immune system [48,49]. This mode of cell death is triggered by activation of GTPase RhoA, followed by MAPK phosphorylation, which reduces SLC9A1 expression and activity [49]. Downregulation of SLC9A1, associated with methylated DNA sequence, is one of established risk factors in cardiovascular diseases [48]. Similarly to *RUNX3*, it is commonly targeted by TGF-β and Notch signals [50].

### 4.4. Mitochondrial Dynamics

Since our gene expression analysis implied that mitochondrial dynamics were related to *RUNX3* expression, we examined mitochondria-mediated pathways. The findings suggested repression of *RUNX3* via coiled-coil-helix-coiled-coil-helix domain containing 1 (*CHCHD1)* expression in circulating CD14+ cells. This negative correlation may support previous observations on mitochondrial translational machinery [51] involved in RUNX3-induced apoptosis [52]. Role of *CHCHD1* for human pathologies was explicated by placing this gene in the Hippo signaling pathway. Expression of *CHCHD1* was associated with yes-associated protein 1 (*YAP1*), which is one of the crucial downstream effectors of the Hippo pathway [51]. RUNX3 showed an ability to replace the binding partner of YAP. Destabilization of the YAP-TEAD bound was conditioned through the YAP phosphorylation, which facilitated the creation of the YAP-TEAD-RUNX3 ternary complex [53]. Therefore, inhibition of TEAD, induced by RUNX3, might predominate YAP-activating signaling and limit gastrointestinal tumorigenesis [52,53].

### 4.5. Particles Uptake and Trafficking

Again, we applied immune-centered DRAIMI as an auxiliary method of transcriptomic analysis to gain insight into monocyte metabolism. We found an interaction between the expression of *RUNX3* and very low-density lipoprotein receptor (*VLDLR*) among the five tops of 1000 differentially expressed transcripts from both MESA and CEDAR datasets. Knowing that monocytes are exposed to lipid-rich lipoproteins in the bloodstream, our protein–protein network suggests RUNX3 involvement in lipid deposition. White blood cells constantly exposed to LDL or VLDL acquire an atherogenic phenotype, presented by highly expressed VLDLR mRNA and low VLDL-C [54]. Despite this feature, monocytes differ from other WBCs. Lack of active gene expression in some metabolic pathways [55] was found to supply monocytes with distinct chemotactic properties, lipid metabolism, and gene expression profile in response to lipid vesicles [56]. Neutral lipid loading, under exposure to low-density lipoproteins, impairs monocyte responsiveness to chemotactic stimuli. Previous studies noted that defect in chemotaxis occurred through RHOA inactivation [57]. WNT signaling is transduced to β-catenin and RHOA signals, maintaining embryogenesis and tissue homeostasis. Expression of *RUNX3* enables RUNX3/β-catenin complex formation, thus attenuating cascades. Inhibitors of the WNT pathway, including *RUNX3*, are frequently methylated and therefore inactivated in pathologies such as acute myeloid leukemias [58], or gastrointestinal cancers [59,60].

### 4.6. Immune Correlates

Several important immune genes were found among the moderately strong positive correlates of *RUNX3* in monocytes. Their roles related to cytokine signaling via molecules such as interleukin 10 receptor subunit alpha (*IL10RA*), inhibitor of nuclear factor kappa B kinase regulatory subunit gamma (*IKBKG*), interleukin 4 receptor (*IL4R*), Janus kinase 1 (*JAK1*). Interferon signaling was highlighted by the presence of interferon response factors 1 and 7 (*IRF1*, *IRF7*), the antiviral protein 2′-5′-oligoadenylate synthetase 2 (*OAS2*), and tyrosine kinase 2 (*TYK2*). They also related to IL1 signaling: possible recruitment of IKK complex by interleukin 1 receptor associated kinase 1 (*IRAK1*) and interleukin 18 binding protein (*IL18BP*) interference with signaling via IL18 (which belongs to IL1 family). One of the top positive correlates of *RUNX3* was C-type lectin domain containing 16A (*CLEC16A*) of a group of proteins related to (auto)immunity and IBD [61].

### 4.7. Generalization and Limitations

This study analyzed transcript abundance, which does not necessarily reflect protein abundance. High expression of *RUNX3* might not correlate with high RUNX3 activity as a transcription factor. Yet, *RUNX3* expression was analyzed within the context of the entire transcriptome, from which inferences could be made about potential interactions with other genes. Clinical data were not considered in this cross-sectional bioinformatic analysis of monocyte transcriptional states. The monocyte transcriptomes came from mixtures of many cells, but their variability was exploited to gain functional insights. One could argue that immunomagnetic separation is not precise enough to provide pure CD14+ cells. However, at this scale, only data from immunomagnetically separated cells are available. Single-cell sequencing would not permit to obtain the same knowledge as this study provides, because of overall very low expression levels when individual cells are analyzed, excluding genes with lower mean expression. Because single-cell sequencing has powerful clustering capacities, it would be interesting to investigate monocyte transcriptomes in this manner and to integrate the knowledge with bulk transcriptomes from monocytes sorted by flow cytometry. Protein–protein interaction networks were used to provide knowledge of relationships between genes, on which the results of transcriptomic analyses were overlaid, with the aim of generating new biological hypotheses. Joining data from such external sources (including other omics) is not meant to imply direct relationships, but to strengthen the conceptual analysis, and it is also a common practice in omics research. There is no multi-omics integration in this study, which would cover the genome and the epigenome. Finally, it should be added that this study intersects the results from two independent cohorts to improve generalizability and reduce false positive rate at some potential cost to the true positive rate.

## 5. Conclusions

In summary, our integrated, cross-sectional transcriptomic analysis in two large, independent datasets (MESA, CEDAR) revealed several *RUNX3*-associated genes and pathways in monocytes. *RUNX3*-correlated genes refer to fundamental processes such as transcription, cytoskeleton rearrangement, and signal transduction. This broad importance of *RUNX3* in CD14+ cells, as inferred from gene expression profiling, extends to immune regulation, especially tyrosine kinases and rho GTPases. Many of identified genes were linked to RUNX3-related mechanisms for the first time and may lead to further experiments to generate omics and functional data in immune cells.

## Figures and Tables

**Table 1:** Genes most strongly positively and negatively correlated with *RUNX3* in monocyte expression profiles from MESA and CEDAR studies. Mean r correlation coefficient value is used to highlight main results that are most consistent across both cohorts. FDR–false discovery rate.

Gene	r_mean_	r_MESA_	p_FDR-MESA_	r_CEDAR_	p_FDR-CEDAR_	Gene Name
Positive correlates of *RUNX3*						
*EVL*	0.75	0.65	5.37 × 10^−140^	0.86	5.52 × 10^−80^	Enah/Vasp-Like
*ARHGAP17*	0.74	0.69	1.13 × 10^−169^	0.79	9.20 × 10^−59^	Rho GTPase-Activating Protein 17
*DNMT1*	0.74	0.69	1.10 × 10^−169^	0.79	1.67 × 10^−58^	DNA Methyltransferase 1
*RAPGEF1*	0.73	0.69	1.66 × 10^−168^	0.76	2.09 × 10^−51^	Rap Guanine Nucleotide Exchange Factor 1
*CLEC16A*	0.72	0.67	3.51 × 10^−154^	0.78	2.27 × 10^−55^	C-Type Lectin Domain Containing 16A
*ARHGEF18*	0.72	0.67	6.61 × 10^−152^	0.77	1.14 × 10^−53^	Rho/Rac Guanine Nucleotide Exchange Factor 18
*SIPA1*	0.71	0.64	9.91 × 10^−137^	0.79	1.08 × 10^−57^	Signal-Induced Proliferation-Associated 1
*GLG1*	0.71	0.65	4.24 × 10^−143^	0.77	2.45 × 10^−53^	Golgi Glycoprotein 1
*HNRNPUL2*	0.71	0.68	7.38 × 10^−161^	0.74	1.31 × 10^−47^	Heterogeneous Nuclear Ribonucleoprotein U Like 2
*FNBP1*	0.71	0.66	1.21 × 10^−146^	0.76	1.56 × 10^−51^	Formin-Binding Protein 1
Negative correlates of *RUNX3* (most significant at the bottom)						
*C11ORF54*	−0.50	−0.47	2.07 × 10^−67^	−0.53	1.18 × 10^−20^	Chromosome 11 Open Reading Frame 54
*LOC100132510*	−0.50	−0.43	2.56 × 10^−55^	−0.57	1.22 × 10^−24^	-
*HNMT*	−0.51	−0.44	3.96 × 10^−58^	−0.58	4.75 × 10^−25^	Histamine N-Methyltransferase
*PRKAG1*	−0.52	−0.51	1.81 × 10^−79^	−0.54	2.16 × 10^−21^	Protein Kinase AMP-Activated Non-Catalytic Subunit Gamma 1
*TMEM120A*	−0.54	−0.43	1.97 × 10^−54^	−0.64	2.94 × 10^−32^	Transmembrane Protein 120A
*CHCHD1*	−0.54	−0.55	1.36 × 10^−93^	−0.53	1.72 × 10^−20^	Coiled-Coil-Helix-Coiled-Coil-Helix Domain Containing 1
*ELMOD2*	−0.54	−0.53	8.30 × 10^−86^	−0.56	6.61 × 10^−23^	ELMO Domain Containing 2
*LOC100129118*	−0.55	−0.56	2.50 × 10^−97^	−0.54	4.80 × 10^−21^	-
*TBC1D7*	−0.55	−0.48	1.36 × 10^−69^	−0.62	7.81 × 10^−30^	TBC1 Domain Family Member 7
*C2ORF76*	−0.57	−0.55	8.70 × 10^−94^	−0.58	1.31 × 10^−25^	Chromosome 2 Open Reading Frame 76

**Table 2:** Biological process gene ontology terms from GSEA analysis of top 100 transcripts positively and negatively correlated with *RUNX3*.

Ontology Term	FDR q-Value
Transcripts positively correlated with *RUNX3*	
mRNA metabolic process	7.94 × 10^−9^
Regulation of mRNA metabolic process	5.87 × 10^−6^
Small GTPase-mediated signal transduction	5.87 × 10^−6^
Cytoskeleton organization	1.42 × 10^−5^
Chromosome organization	2.62 × 10^−5^
Histone modification	3.19 × 10^−5^
Positive regulation of nucleobase containing compound metabolic process	9.20 × 10^−5^
Positive regulation of RNA metabolic process	9.20 × 10^−5^
Establishment of RNA localization	9.20 × 10^−5^
Chromatin organization	1.07 × 10^−4^
Transcripts negatively correlated with *RUNX3*	
Protein insertion into mitochondrial inner membrane	6.40 × 10^−3^
Mitochondrial transport	6.40 × 10^−3^
Intracellular transport	4.13 × 10^−2^
Establishment of protein localization to mitochondrial membrane	4.13 × 10^−2^

**Table 3:** Genes selected by random forest models as most related to *RUNX3* expression. Random forests may be able to capture potential nonlinear effects.

MESA		CEDAR	
Gene	Importance	Gene	Importance
*SLC9A1*	100.00	*RIOK1*	100.00
*C14ORF43*	98.28	*ADAR*	96.82
*FAM193A*	96.45	*CNIH4*	94.51
*BICD2*	93.41	*FRMD8*	93.03
*CCDC88A*	90.24	*SLC35C2*	89.46
*ASAP1*	88.84	*LARP4B*	88.81
*ARHGAP17*	83.68	*BOP1*	85.59
*ARHGEF18*	82.81	*C14orf142*	83.66
*FGR*	81.21	*MID2*	81.05
*TNK2*	80.50	*ADD3*	79.50
*SFRS2IP*	79.80	*RBL2*	78.56
*LOC100130914*	76.74	*SAFB2*	78.30
*SCAPER*	75.22	*CHD1L*	76.89
*MYPOP*	72.76	*DIAPH2*	76.86
*PRKCD*	70.49	*IFT122*	73.77
*CEP110*	69.97	*NARG2*	72.48
*PRR13*	69.31	*ARHGEF18*	72.42
*TNFRSF1B*	69.29	*PRKCH*	71.86
*RHBDF2*	68.89	*LAMP1*	69.39
*ZDHHC8*	68.23	*CCDC130*	69.07

**Table 4:** Intersection of genes from *RUNX3* expression-predicting random forest models in MESA and CEDAR. Only genes with importance > 40 in both datasets were intersected.

Gene	Importance in MESA	Importance in CEDAR
*ARHGEF18*	82.81	72.42
*SLC9A1*	100.00	42.47
*ARHGAP17*	83.68	50.20
*TACC1*	40.01	59.56

**Table 5:** Results of immune-centered DRAIMI analysis of top vs. bottom 10% of MESA and CEDAR samples by *RUNX3* expression. Only genes identified in analyses from both MESA and CEDAR are included. Intermediary ratios from DRAIMI are shown, where higher values indicate greater relationship with *RUNX3* expression (the number of identified interactions among top 1000 differentially expressed transcript ratios vs. the total number of interactions in the network for given entity).

Gene	MESA	CEDAR	Mean	Gene Name
*NGF*	0.24	0.23	0.23	Nerve Growth Factor
*CBL*	0.27	0.12	0.20	Cbl Proto-Oncogene
*RAP1B*	0.28	0.10	0.19	RAP1B, Member Of RAS Oncogene Family
*VLDLR*	0.29	0.06	0.17	Very Low Density Lipoprotein Receptor
*MET*	0.17	0.11	0.14	MET Proto-Oncogene, Receptor Tyrosine Kinase
*FRS2*	0.15	0.12	0.13	Fibroblast Growth Factor Receptor Substrate 2
*RAP1A*	0.17	0.08	0.12	RAP1A, Member Of RAS Oncogene Family
*HLA-E*	0.05	0.19	0.12	Major Histocompatibility Complex, Class I, E
*PTPN11*	0.14	0.09	0.12	Protein Tyrosine Phosphatase Non-Receptor Type 11
*PSMD1*	0.17	0.05	0.11	Proteasome 26S Subunit, Non-ATPase 1
*VAMP8*	0.17	0.04	0.11	Vesicle Associated Membrane Protein 8
*RHOA*	0.07	0.14	0.10	Ras Homolog Family Member A
*LCK*	0.12	0.08	0.10	LCK Proto-Oncogene, Src Family Tyrosine Kinase
*FYN*	0.11	0.08	0.10	FYN Proto-Oncogene, Src Family Tyrosine Kinase
*GAB1*	0.14	0.05	0.09	GRB2 Associated Binding Protein 1
*PSMB8*	0.06	0.13	0.09	Proteasome 20S Subunit Beta 8
*ACTG1*	0.04	0.15	0.09	Actin Gamma 1
*BRAF*	0.13	0.04	0.09	B-Raf Proto-Oncogene, Serine/Threonine Kinase
*KPNB1*	0.12	0.05	0.09	Karyopherin Subunit Beta 1
*YES1*	0.11	0.06	0.08	YES Proto-Oncogene 1, Src Family Tyrosine Kinase

## Data Availability

The data are publicly available from the original study—the Multi-Ethnic Study of Atherosclerosis (Gene Expression Omnibus GSE56047)—and the Correlated Expression and Disease Association Research (BioStudies E-MTAB-6667).

## Supplementary Materials

The following supporting information can be downloaded at https://www.mdpi.com/article/10.3390/biomedicines11061698/s1. Figure S1: Clustering of top 100 genes from immune-oriented DRAIMI analysis together with RUNX3, which was based on STRING protein–protein interaction networks; Table S1: Intersection of MESA–CEDAR correlation analysis; Table S2: Variables included in random forest models from MESA and CEDAR studies, ranked by importance (as measured using impurity); Table S3: DRAIMI results.
