# Supplementary figures and images for: Transcriptomic Context of RUNX3 Expression in Monocytes: A Cross-Sectional Analysis
Source: Biomedicines. 2023 Jun 13;11(6):1698. doi: 10.3390/biomedicines11061698 (PMC10296263; doi:10.3390/biomedicines11061698)

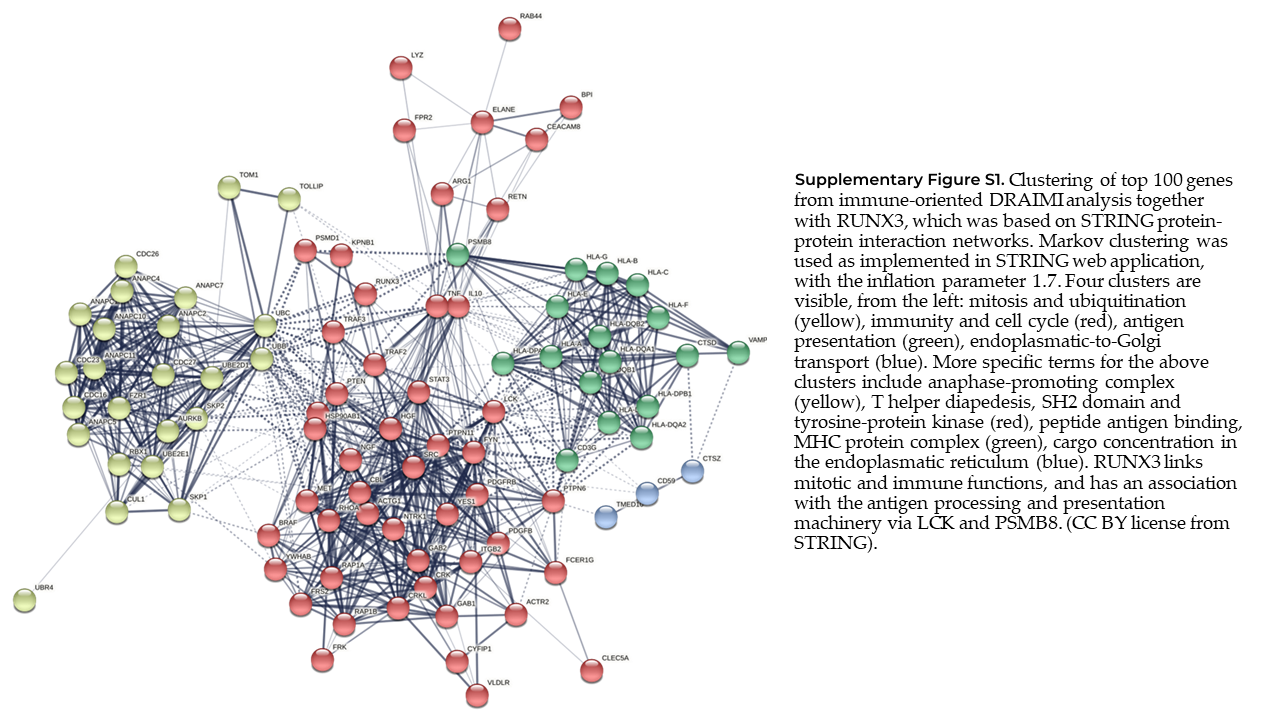

Supplement: Supplementary file 1 [file biomedicines-11-01698-s001.zip › Figure S1 Clustering from DRAIMI analysis.png]
